# Mixed Pro- and Anti-Oxidative Effects of Pomegranate Polyphenols in Cultured Cells

**DOI:** 10.3390/ijms151119458

**Published:** 2014-10-27

**Authors:** Francesca Danesi, Paul A. Kroon, Shikha Saha, Dario de Biase, Luigi Filippo D’Antuono, Alessandra Bordoni

**Affiliations:** 1 Department of Agri-Food Sciences and Technologies, University of Bologna, Piazza Goidanich, 60-47521 Cesena (FC), Italy; E-Mails: filippo.dantuono@unibo.it (L.F.D.); alessandra.bordoni@unibo.it (A.B.); 2 Food & Health Programme, Institute of Food Research, Norwich Research Park, Norwich NR4 7UA, UK; E-Mails: paul.kroon@ifr.ac.uk (P.A.K.); shikha.saha@ifr.ac.uk (S.S.); 3 Department of Experimental, Diagnostic and Specialty Medicine, University of Bologna, Via Altura, 3-40139 Bologna (BO), Italy; E-Mail: dario.debiase@unibo.it

**Keywords:** pomegranate, punicalagin, oxidative stress, antioxidant defenses, bioactives, HepG2 cells

## Abstract

In recent years, the number of scientific papers concerning pomegranate (*Punica granatum* L.) and its health properties has increased greatly, and there is great potential for the use of bioactive-rich pomegranate extracts as ingredients in functional foods and nutraceuticals. To translate this potential into effective strategies it is essential to further elucidate the mechanisms of the reported bioactivity. In this study HepG2 cells were supplemented with a pomegranate fruit extract or with the corresponding amount of pure punicalagin, and then subjected to an exogenous oxidative stress. Overall, upon the oxidative stress the gene expression and activity of the main antioxidant enzymes appeared reduced in supplemented cells, which were more prone to the detrimental effects than unsupplemented ones. No differences were detected between cells supplemented with the pomegranate juice or the pure punicalagin. Although further studies are needed due to the gaps existing between *in vitro* and *in vivo* studies, our results suggest caution in the administration of high concentrations of nutraceutical molecules, particularly when they are administered in concentrated form.

## 1. Introduction

The pomegranate (*Punica granatum* L.) tree, which is said to have flourished in the Garden of Eden, has been extensively used as a folk medicine in many cultures, especially in Eastern and Middle Eastern countries where it is cultivated in large amounts. Pomegranate fruits are widely consumed both fresh and in processed forms as juices, jams, jellies, and wines [1,2]. The fruit consists of three parts: seeds (about 3% of the fruit weight), juice (about 30% of the fruit weight), and peels, which also include the interior network of membranes [3]. The fresh juice contains 85% water, 10% total sugars, 1.5% pectin, ascorbic acid, and polyphenolic compounds. The soluble polyphenol content varies within the limits of 0.2% to 1.0%, and includes mainly ellagitannins (as punicalagins), ellagic acids, anthocyanins, flavonols, flavan-3-ols, and flavones [1]. Among the great variety of bioactives present in the pomegranate fruit, phenolic compounds are considered the main one responsible for most of the health benefits, that in the past have been ascribed mainly to the antioxidant potential of these components [4,5].

The health effects of the whole fruit, as well as its juices and extracts, have been studied in relation to a variety of chronic diseases, and pomegranate has gained widespread popularity as a functional food and nutraceutical source. Although there are many studies in the literature on the preventive role of pomegranate in the metabolic syndrome (reviewed in [6]), obesity (reviewed in [7]), hypertension and cardiovascular disease (reviewed in [8]), and other diseases [9], the Panel on Dietetic Products, Nutrition and Allergies of the European Food Safety Authority (EFSA) concluded that a cause and effect relationship between the consumption of pomegranate fruit or juice and the claimed health effects has not been established yet.

Among the main gaps to be filled, there is a lack of substantiated mechanisms of action, that could be related not only to a direct scavenging activity of pomegranate components, but also to the modulation of antioxidant and detoxification enzymes, modulation of cell signaling and gene expression, and other cellular effects [10,11,12]. In addition, great differences in phenols contents and antioxidant activities related to the different cultivar, growing conditions, and/or processing have been demonstrated in different fruits and juices [13], thus possibly influencing the final effect of pomegranate consumption. Hence, in the attempt to unravel the mechanism of action of pomegranate (POM) and its components, in the present study we focused on an extract obtained from a specific pomegranate cultivar grown in the Kakheti region (Georgia). The study herein reported is part of the EU funded project (BaSeFood “Sustainable exploitation of bioactive components from the Black Sea Area traditional foods”; EC Contract no: FP7-KBBE-227118), and this particular pomegranate cultivar, grown in a specific region of the Black Sea Area, was chosen to accomplish the main objective of the project itself, *i.e.*, the reevaluation of the Black Sea Area’s traditional plant foods, like the Azerbaijani pomegranate.

Georgian fruits were characterized by HPLC, and its potential bioactivity was evaluated in HepG2 cells in basal condition and upon exposure to a pro-oxidative agent. The gene expression and the activity of the main antioxidant enzymes (superoxide dismutase—SOD; catalase—CAT; and glutathione peroxidase—GPX) were determined in both basal and oxidative condition, and the protective effect of the extract evaluated. Furthermore, in parallel experiments cells were supplemented with an amount of punicalagin (PUN) corresponding to its concentration in the extract allowing us not only to discriminate between the contribution of the main phenolic component and the synergistic effect of all the phytochemicals present in the POM, but also to compare our results to other studies on the basis of the PUN concentration in the considered pomegranate extracts.

## 2. Results and Discussion

The HPLC analysis of POM (Table 1) showed a pomegranate-specific fingerprint of polyphenol classes, which include PUN (isomers A and B), ellagic acid, and a large pool of hydrolysable tannins or polyphenols.

**Table 1:** POM (pomegranate) phenolic profile. The concentration of the main phenolic compounds, as detected by HPLC, is expressed in mg analyte/g.

Compound	mg/g
Punicalagin (A + B)	1.830 ± 0.01
Free ellagic acid	0.048 ± 0.00
Cyanidin-3,5-di-*O*-glucoside	0.126 ± 0.01
Delphinidin-3-*O*-glucoside	0.173 ± 0.00
Cyanidin-3-*O*-glucoside	0.118 ± 0.00
(–)-Epicatechin	0.018 ± 0.00

**Figure 1:**
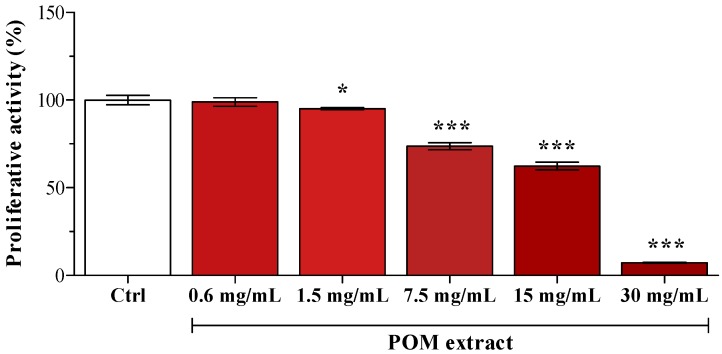
Cell viability after supplementation with different POM concentrations. Data are expressed as the percentage of the value obtained in unsupplemented cells (Ctrl), assigned as 100%. Statistical analysis was carried out by one-way ANOVA (*p* < 0.001) using Dunnett’s post test (* *p* < 0.05, *** *p* < 0.001 *vs.* Ctrl cells).

In preliminary experiments, POM cytotoxicity was assessed by supplementing HepG2 cells with serial dilutions (from 0.6 to 30 mg/mL of medium). As reported by Karaaslan *et al.* [14], pomegranate extract evidenced a strong anti-proliferative activity (Figure 1); consequently, POM concentration used in the following experiments was the highest one evidenced as not cytotoxic (0.6 mg/mL). PUN final concentration in the medium after the addition of 0.6 mg/mL POM was 1 µM, and some cells were supplemented with 1 µM PUN.

The *tert*-butyl hydroperoxide (*t*-BOOH) concentration able to induce an oxidative stress in HepG2 cells was also determined in preliminary experiments measuring cell viability in unsupplemented cells by the MTT assay (data not shown), and the 300 µM *t*-BOOH concentration, resulting in an about 15% reduction of viability, was chosen for subsequent experiments.

**Figure 2:**
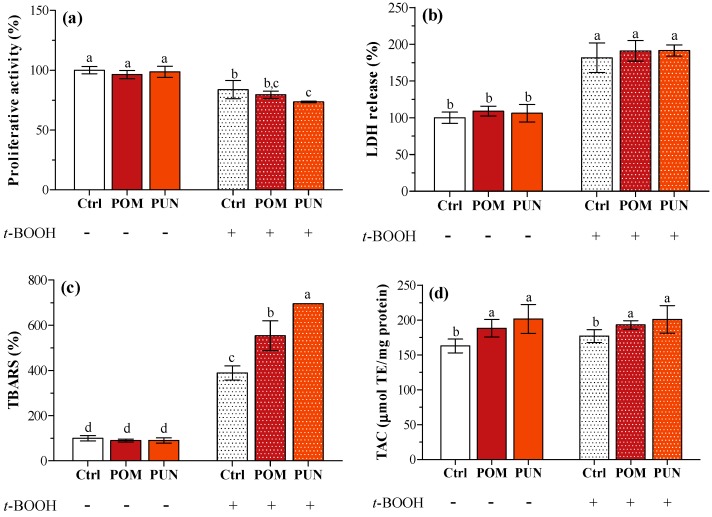
(**a**) Cell proliferative activity; (**b**) LDH release; (**c**) TBARS concentration; and (**d**) TAC of unsupplemented and supplemented cells in basal and stressed conditions. Data in panels **a**, **b** and **c** are expressed as the percentage of the value obtained in not stressed Ctrl cells (assigned as 100%), while TAC (panel **d**) is expressed as micromoles of trolox equivalent (TE) per mg protein. Statistical analysis was carried out by one-way ANOVA (panels **a**, **b**, and **c** *p* < 0.001; panel **d** *p* < 0.01) using Tukey’s HSD test. Different letters indicate statistical significance (at least *p* < 0.05).

In basal condition, no differences in proliferative activity, lactate dehydrogenase (LDH) release, and thiobarbituric reactive substances (TBARS) concentration between unsupplemented and supplemented cells were detected (Figure 2a–c, respectively). Compared to basal condition, the exposure to *t*-BOOH caused a significant decrease in cell proliferative activity (Figure 2a), and a significant increase in LDH leakage (Figure 2b) and TBARS concentration in the medium (Figure 2c) independent of supplementation. As observed in a previous study [13], POM and PUN supplementation increased cellular total antioxidant capacity (TAC); a significant increase in cytosolic TAC was also observed in oxidative condition (Figure 2d).

The protective effect of pomegranate is usually ascribed to the antioxidant activity of its components. It is well known that the antioxidant action of many bioactive molecules is not limited to ROS scavenging, and includes the modulation of cell signaling, gene expression and activity of antioxidant enzymes [12]; the measurement of the cytosolic TAC cannot discriminate between a direct scavenging activity and the modulation of enzyme antioxidant defenses, so the expression and activity of SODs, CAT and GPX was also evaluated.

**Figure 3:**
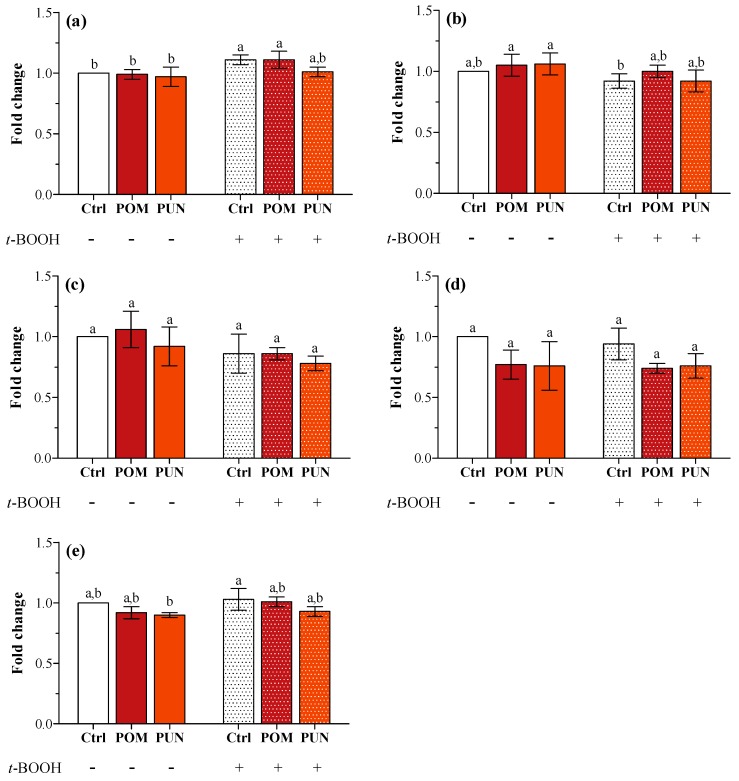
(**a**) *SOD1*; (**b**) *SOD2*; (**c**) *CAT*; (**d**) *GPX1*; and (**e**) *GPX4* gene expression in unsupplemented and supplemented cells in basal and stressed conditions. Data are expressed as the mean fold change of relative expression compared to not stressed Ctrl cells, normalized to one. Statistical analysis was carried out by one-way ANOVA (panel **a** *p* < 0.001, panels **b** and **e** *p* < 0.01; panels **c** and **d** n.s.) using Tukey’s HSD test. Different letters indicate statistical significance (at least *p* < 0.05).

In basal condition, neither POM nor PUN supplementation modulated the expression of the genes encoding for SOD1 and 2, CAT, GPX1 and 4 (Figure 3a–e, respectively). The exposure to *t*-BOOH did not cause any modification in the expression of the tested genes, apart from an increase in *SOD1* transcription in Ctrl and POM supplemented stressed cells.

**Figure 4:**
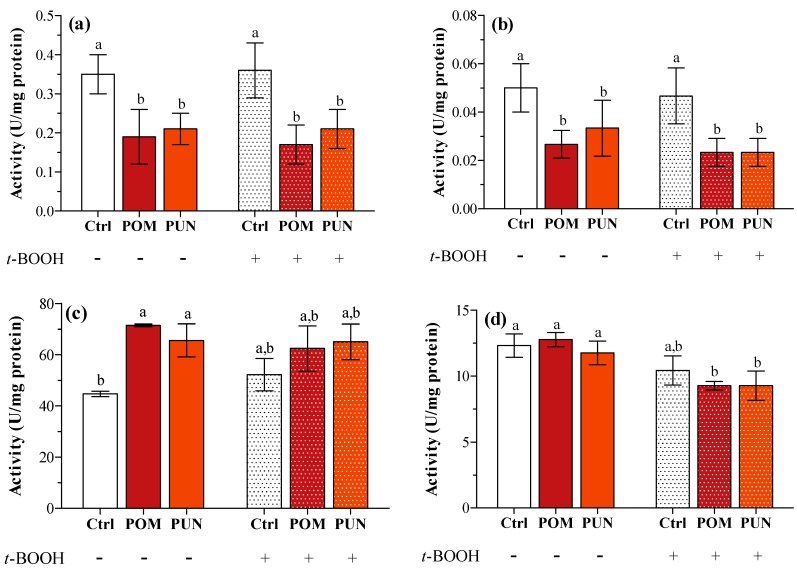
(**a**) SOD1; (**b**) SOD2; (**c**) CAT; and (**d**) GPX enzyme activity in unsupplemented and supplemented cells in basal and stressed conditions. Data are expressed as units (U) per mg protein. Statistical analysis was carried out by one-way ANOVA (panels **a**, **b** and **c** *p* < 0.01; panel **d** *p* < 0.001) using Tukey’s HSD test. Different letters indicate statistical significance (at least *p* < 0.05).

This lack of any effect on gene expression could be explained considering a very short-term effect on transcription, not detectable after 24 h supplementation and/or 3 h exposure to the oxidative stress. A short-term effect on transcription can anyway be detected in later time when measuring protein activity; in fact, a significant decrease in SOD1 and 2 activity was clearly detected in POM and PUN supplemented cells in both basal and stressed conditions (Figure 4a,b, respectively), and CAT activity significantly increased in supplemented cells in basal condition (Figure 4c). In basal condition GPX activity was similar in all groups, and it significantly decreased after *t*-BOOH treatment in POM and PUN supplemented cells (Figure 4d).

An almost similar pattern of results was found in mice by Faria *et al.* [10], who evidenced a decreased SOD, CAT, and GPX activity after treatment with pomegranate juice, without any variation in mRNA expression. The Authors explained their results considering that the increased concentration of exogenous scavenging molecules due to pomegranate juice supplementation could have reduced the concentration of superoxide anion. This in turn could have decreased the activity of SOD, which catalyzes the dismutation of superoxide anion producing H_2_O_2_; the consequent reduction of this ROS could have resulted in a decreased CAT and GPX activity, which catalyzes the reduction of hydroperoxides. The Authors concluded that their results were compatible with a protective effect of pomegranate juice against systemic oxidative stress in mice, since endogenous antioxidant defenses were lowered as they are no longer required to act on an organism supplied with generous amounts of exogenous antioxidants.

This conclusion does not fit with the present study, not only because CAT activity increased in POM and PUN supplemented cells in basal condition, but mainly because the supplementation did not preserved cells from the reduction in vitality, membrane damage, and the increase in lipid peroxidation induced by *t*-BOOH exposure. Notwithstanding the higher cytosolic TAC in supplemented cells, in stressed condition POM and PUN seemed to reduce the overall defenses related to the activity of the main antioxidant enzymes. This could explain the failure to protection from the consequences of the oxidative stress.

The lack of effectiveness of POM and PUN supplementation could be explained in different ways. First, many studies reporting a protective effect have been performed *in vivo*, and it is known that polyphenols are metabolized to ellagic acid and urolithins, suggesting that the bioactive compounds that provide *in vivo* antioxidant activity may not be the same as those present in the whole food. The Authors are aware that the use of POM and PUN represents a limitation of the present research, since polyphenols are rather unstable in cell medium and native polyphenols in pomegranate extract are unlikely metabolized *in vitro* the same way as they are *in vivo* [15]. Nevertheless Larrosa *et al.* [16] have demonstrated that PUN is hydrolyzed in the cell medium yielding ellagic acid even in the absence of cells. Therefore it can be speculated that the observed effects—of the same extent in POM and PUN supplemented cells—are in part due to the derived-metabolite ellagic acid.

As well, the amount of POM and PUN used for cell supplementation could represent an important variable in the determination of the final effect. In the work by Di Nunzio *et al.* [13], HepG2 cells were supplemented with different concentrations of pomegranate juice from different cultivar, and stressed with H_2_O_2_. All but one of the juices protected cells from the reduction in cell viability and the increase in LDH leakage induced by the oxidative stress. Pomegranate juices used in that study have a different PUN content and, according to the reported HPLC composition the amount of PUN supplemented to HepG2 cells, was between 0.03 and 0.23 µg/mL medium, and 0.46 µg/mL medium in the non-protective juice. In the present study, HepG2 cells were supplemented with 1.09 µg PUN/mL medium both as fruit extract or as pure PUN. It is therefore evident that the amount of PUN is critical in determining the final effect, high amount of the compound being even deleterious to cells when exposed to an exogenous oxidative stress.

Last but not least, despite the wide use of cell cultures for the determination of bioactives effectiveness and mechanism of action, an important scientific gap is seldom considered. Cell cultures represent a close system, and their direct and continuous exposure to bioactives could alter the cell response, inducing paradoxical effects, due to the lack of the continuous detoxification and clearance of compounds occurring in the whole body. This effect should be considered even using concentrations that are physiological *in vivo* and have been tested as not cytotoxic *in vitro*. Although the concentration of POM and PUN we used in the present study is far below the polyphenol and ellagitannin metabolites concentrations found in human plasma [17], and it was assessed as not cytotoxic in basal condition, a paradoxical effect was observed in oxidative condition, exacerbating the consequences of the exogenous stress.

## 3. Experimental Section

### 3.1. Materials

All chemicals, reagents, and solvents were purchased from Sigma-Aldrich Co. (St. Louis, MO, USA) unless otherwise stated. Punicalagin was dissolved in ethanol at a concentration of 1 mg/mL; other solutions were prepared using ultrapure water (Milli-Q; Millipore; Bedford, CT, USA).

### 3.2. Pomegranate Fruit Extract

Pomegranate fruit extract (POM) was produced from 30 fresh pomegranate fruits (*Punica granatum* L.) grown in Kakheti region (Georgia). The procedure used to extract the polyphenols from pomegranate fruit used the widely reported hot methanol method and has been described elsewhere [18]. Briefly, the arils were removed and then blended in a food processor. After blending, the aril mix was decanted into a glass container, mixed with hot (65–70 °C) methanol (3:10 *v*/*v* aril mix/methanol), and incubated at 65–70 °C for 20 min before filtering and centrifuging the alcoholic extract. After centrifugation, the crude extract was evaporated to dryness using a rotary evaporator and then sealed, prior to storage at −20 °C.

The methanolic extract was analyzed by HPLC-DAD/MS using either positive or negative polarity modes as previously reported [18,19].

POM was dissolved in ultrapure water at the concentration of 60 mg/mL; clear solution was prepared by gentle heating (at 40 °C) and stirring. The stock solution was filtered through sterile 0.22 µm, aliquoted and stored at −20 °C until further use.

### 3.3. Cell Culture

HepG2 cells were grown in Dulbecco’s modified Eagle medium (DMEM) (Lonza; Basel, Switzerland) supplemented with 10% fetal bovine serum (FBS), 100 U/mL penicillin, and 100 µg/mL of streptomycin. Cells were seeded at ≈1,000,000 cells per well in six-well plates. Twenty-four hours after seeding, the culture medium was replaced with fresh one containing 0.6 mg/mL POM or 1 μM punicalagin (PUN, isolated from pomegranate; Sigma-Aldrich Co.), corresponding to the amount of PUN supplemented with the POM extract. Unsupplemented cells (Ctrl) received fresh medium with no added extract. Twenty-four hours later, in some cells the medium was discarded and modified RPMI-1640 medium without phenol red, containing 300 µM of *tert*-butyl hydroperoxide (*t*-BOOH), was added to induce an oxidative insult (stressed condition), while other cells received RPMI-1640 medium without *t*-BOOH (basal condition). After 3 h, media were removed and collected for further analysis. Cells were washed twice with 1 mL of ice-cold 0.9% NaCl, and further analysis performed.

### 3.4. Cytotoxicity Testing

Three commonly used *in vitro* test systems monitoring different cytotoxicity endpoints were applied: cellular metabolic activity (MTT assay) [20], plasma membrane integrity (lactate dehydrogenase—LDH assay) [21], and lipid peroxidation (assay for thiobarbituric reactive substances—TBARS) [22]. These assays were carried out as described previously [19,23].

### 3.5. Total Antioxidant Capacity (TAC)

TAC was determined in cell lysates obtained as previously described [19]. TAC was determined using the method of Re *et al.* [24] based on the capacity of antioxidant molecules to reduce the radical cation of 2,2'-azinobis(3-ethylbenzo-6-thiazoline-6-sulfonic acid) (ABTS), determined by the discoloration of ABTS^•+^, and measured as the quenching of the absorbance at 734 nm. Values obtained for each sample were compared to the concentration-response curve of the standard trolox solution. Cellular TAC was expressed as micromoles of trolox equivalent (TE) per mg protein.

### 3.6. Real-Time PCR

Cells were lysed directly in the culture dish and total RNA was isolated using the RNeasy kit (Qiagen; Hamburg, Germany) according to manufacturer’s protocol. The quantity and quality of RNA were assessed using the NanoDrop ND-2000 spectrophotometer (Thermo Fisher Scientific; Wilmington, DE, USA). Samples were frozen at −80 °C until further use.

One μg RNA was reverse transcribed with oligo-dT and random primers to create cDNA using the QuantiTect reverse transcription kit (Qiagen). Two independent run-off transcription assays were performed, and transcripts were pooled together.

cDNA, obtained as described above, was subjected to quantitative real-time PCR analysis using the Rotor-Gene 6000 (Corbett Research; Mortlake, Australia) detection system, according to QuantiTect SYBR Green RT-PCR kit (Qiagen). The reaction consisted of 25 µL, containing 12.5 µL Sybr master mix 2X, 1 µL sense primer (5 µmol/L), 1 µL antisense primer (5 µmol/L), 9.5 µL RNase-free H_2_O, and 1 µL cDNA. Samples were run in duplicate using the following program: initial denaturation at 95 °C for 15 min, followed by 40 cycles of 94 °C for 15 s, 55 °C for 30 s, and 72 °C for 30 s.

The reference genes used in our study (*ACTB*, *GAPDH*, *HMBS*, and *SDHA*) were selected based on previously published literature [25]. Primer sequences for these genes were chosen from the RTPrimerDB online real-time PCR primer database [26,27]. Specific primers for target genes were designed using the publicly available web-based Primer3 program [28,29]. Each primer set was checked for specificity using the basic local alignment sequence tool (BLAST) against the NCBI database [30,31]. All oligonucleotides used in this study were synthesized by Integrated DNA Technologies (Leuven, Belgium). For sequences of forward and reverse primers, see Table 2.

The specificity of real time RT-PCR products was confirmed in each run by melting curve analysis after the final cycle. Additionally 2% agarose gel containing 0.01% SYBR Safe DNA Gel Stain (Life Technologies Ltd.; Paisley, UK) was performed; band sizes were compared with a molecular weight marker (100 bp DNA Ladder Ready to Load, Solis BioDyne; Tartu, Estonia) to ensure that each was consistent with its predicted size.

Data were analyzed using DataAssist Software version 3.01 (Applied Biosystems; Foster City, CA, USA). Average fold change and standard deviation (SD) were obtained from six biological replicate samples per condition.

**Table 2:** Primer sequences for RT-PCR.

Gene Name	GenBank Accession Number	Primer Sequence	Amplicon Size
*Reference genes*			
*ACTB* β-actin	NG_007992.1	F: ATGTGGCCGAGGACTTTGATT	107 bp
		R: AGTGGGGTGGCTTTTAGGATG	
*GAPDH* glyceraldehyde-3-phosphate dehydrogenase	NG_007073.2	F: AAGGTGAAGGTCGGAGTCAA	108 bp
		R: AATGAAGGGGTCATTGATGG	
*HMBS* hydroxymethylbilane synthase	NG_008093.1	F: ACCAAGGAGCTTGAACATGC	145 bp
		R: GAAAGACAACAGCATCATGAG	
*SDHA* succinate dehydrogenase complex, subunit A	NG_012339.1	F: TGGGAACAAGAGGGCATCTG	86 bp
		R: CCACCACTGCATCAAATTCATG	
*Target genes*			
*CAT catalase*	NG_013339.1	F: ATCCAGAAGAAAGCGGTCAA	133 bp
		R: GAGATCCGGACTGCACAAA	
*GPX1* glutathione peroxidase 1	NG_012264.1	F: AGTTTGGGCATCAGGAGAAC	116 bp
		R: GTTCACCTCGCACTTCTCG	
*GPX4* glutathione peroxidase 4	NC_000019.9	F: GCACATGGTTAACCTGGACA	105 bp
		R: AGGTCGACGAGCTGAGTGTAG	
*SOD1* superoxide dismutase 1, soluble (Cu/Zn-SOD)	NG_008689.1	F: ATGAAGAGAGGCATGTTGGA	112 bp
		R: ATGATGCAATGGTCTCCTGA	
*SOD2* superoxide dismutase 2, mitochondrial (Mn-SOD)	NG_008729.1	F: GTTGGCCAAGGGAGATGTTA	108 bp
		R: TTAGGGCTGAGGTTTGTCCA	

F: forward primer; R: reverse primer.

### 3.7. Antioxidant Enzyme Activity Assays

The activity of the antioxidant enzymes SODs, CAT, and GPX was measured by kits from Cayman Chemical Company (Ann Arbor, MI, USA) using a Tecan Infinite M200 microplate reader (Tecan; Salzburg, Austria). Briefly, after a washing with 0.9% NaCl, cells were harvested in an ice-cold phosphate-buffered saline (PBS). Cell pellets were collected by centrifugation at 1500× *g* for 10 min at 4 °C, and then lysed with a specific ice-cold lysis buffer. Samples for SOD activity were lysed in cold 20 mM HEPES buffer (pH 7.2), containing 1 mM EGTA, 210 mM mannitol, and 70 mM sucrose. Cell lysate were centrifuged at 1500× *g* for 5 min at 4 °C. Samples for CAT and GPX activity were lysed in a cold buffer composed of 150 mM sodium chloride, 1.0% Triton X-100, and 1% protease inhibitor cocktail (Sigma-Aldrich Co.) in 50 mM Tris (pH 7.2). Cell lysate were centrifuged at 10,000× *g* for 15 min at 4 °C.

Protein concentration in the supernatants was determined according to Bradford [32] using the Bio-Rad dye-binding protein assay (Bio-Rad Laboratories; Hercules, CA, USA).

The SOD assay kit utilizes tetrazolium salt to quantifying superoxide radicals generated by xanthine oxidase and hypoxanthine. The absorbance was read at 450 nm. SOD activity was calculated based on the standard curve as units per milligram of protein, with 1 U of SOD defined as the amount of enzyme needed to exhibit 50% dismutation of the superoxide radical. Mn-SOD activity was determined by adding potassium cyanide, which is selective inhibitor of Cu/Zn-SOD, with a final concentration of 3 mM. Cu/Zn-SOD activity was subsequently calculated by the subtraction of Mn-SOD activity from total SOD activity.

CAT activity was tested based on its reaction with methanol in the presence of H_2_O_2_. The formaldehyde produced was measured spectrophotometrically with Purpald, a chromogen that specifically forms a bicyclic heterocycle with aldehydes, which upon oxidation changes from colorless to a purple color. The color change of Purpald was measured at 540 nm. CAT activity was calculated based on the formaldehyde standard curve as units per milligram of protein, with 1 U of CAT defined as the amount of enzyme that produces 1.0 nmol of formaldehyde per minute at 25 °C.

The activity of GPX was determined using the Cayman Chemical kit, with cumene hydroperoxide as substrate. The GPX activity is measured indirectly by a coupled reaction with glutathione reductase. The oxidized glutathione produced when GPX reduces with cumene hydroperoxide is recycled to its reduced state by glutathione reductase and NADPH. The oxidation of NADPH to NADP^+^ is accompanied by a decrease in absorbance at 340 nm, which is directly proportional to the GPX activity in the sample. This assay detects all of the GPX activities in the samples. GPX activity was calculated as units per milligram of protein, with 1 U is defined as the amount of enzyme that oxidizes 1.0 nmol of NADPH to NADP^+^ per minute at 25 °C.

### 3.8. Statistical Analysis

Each experiment was repeated at least twice with different passages of the respective cell line. Resulting data are given as mean ± SD (*n* = 4 to 6). Statistical comparison among treatments was determined by the one-way analysis of variance (ANOVA) with Tukey’s HSD or Dunnett’s test, as indicated in individual figure legends. For all tests, statistical significance was assigned at *p*-value <0.05.

## 4. Conclusions

Polyphenols are supposed to have preventive effects for a variety of chronic pathological conditions, and they are often considered safer and more easily integrated into lifestyle changes than conventional pharmaceutical drugs. However, many studies often report biological effects of food polyphenols without elucidating the underlying molecular, cellular, and physiological mechanisms.

Oxidant properties of polyphenols may be both anti-oxidant and/or pro-oxidant based upon the structure of the particular polyphenols, their concentration, and the cellular redox context [33,34]. With moderate and low polyphenol concentrations, low level oxidative stress may be a beneficial cue to initiate induction of protective anti-oxidant systems and boost immune responses; with higher concentration, or in conditions with increased oxidative stress, polyphenol supplementation may exacerbate the detrimental effect of the stress itself. The biological effects of POM extract and PUN observed in this study could represent evidence that high-dose polyphenols can potentially cause adverse effects through pro-oxidative effects, PUN being the most critical pomegranate component.

Although results obtained *in vitro* cannot be uncritically extrapolated to the *in vivo* situation, this work suggests caution in the use of very high concentrations of nutraceutical molecules, particularly when administered in concentrated form like in fortified, enhanced, or purified foods and/or dietary supplements food.
